# 微小RNA在肺癌中的研究进展

**DOI:** 10.3779/j.issn.1009-3419.2018.09.13

**Published:** 2018-09-20

**Authors:** 磊 脱, 小帅 褚, 莎 沙, 逊 张

**Affiliations:** 1 262500 潍坊，潍坊医学院附属益都中心医院心胸外科 Department of Thoracic and Cardiovascular Surgery, Weifang Yidu Central Hospital, Weifang 262500, China; 2 300051 天津，天津市胸科医院胸外科 Department of Thoracic Surgery, Tianjin Chest Hosptial, Tianjin 300051, China

**Keywords:** 肺肿瘤, 微小RNA, 分子机制, Lung neoplasms, MicorRNA, Molecular mechanism

## Abstract

微小RNA（microRNA, miRNA）是一类大小约为22 nt的非编码RNA，广泛存在于从植物、病毒到人类的各种生物中。microRNA生物学功能广泛，能够与mRNA特异性结合后招募相关RNA酶并导致mRNA降解，从而阻断蛋白编码基因的表达，进而影响其生物学功能。近年来研究发现，microRNA与人类多种恶性肿瘤的发生、发展、侵袭、转移等生物学行为密切相关，并对上述生物学表型起到调控作用。肺癌是发病率最高的恶性肿瘤，其发生发展的确切分子机制并未完全阐明。目前已有研究显示，microRNA在抑癌基因失活、癌基因激活及表观遗传学等方面发挥重要作用。同时，亦有研究报道，microRNA在不同预后的肺癌患者及肺部良性病变中存在明显的差异表达，这种差异表达为microRNA作为肺癌诊断及预生物学标志物提供了可行性。

## Abstract (en)

MicroRNAs (miRNAs) a class of non-coding RNAs about 22 nt in size that are found in a wide range of organisms from plants, viruses to humans. MicroRNA has a wide range of biological functions. It can recruit related RNA enzymes and lead to mRNA degradation after binding to mRNA specificity, thus blocking the expression of protein encoding genes and then affecting their biological functions. In recent years, microRNA has been found to be closely related to the biological behaviors, such as the occurrence, development, invasion and metastasis of multiple human malignant carcinomas, and play a regulatory role in the above biological phenotypes. Lung cancer is the highest incidence of malignancy. The exact molecular mechanism of its occurrence and development has not been fully elucidated. Previous studies have shown that microRNA plays an important role in lung tumor suppressor gene inactivation, oncogene activation and epigenetics. At the same time, there are also reports that there is a significant difference in the expression of microRNA in patients with lung cancer and benign lung diseases. This differential expression provides a basis for the feasibility of microRNA as a diagnostic and pre biological marker for lung cancer.

肺癌是目前临床上较为常见的一类实体恶性肿瘤，流行病学研究显示，2013年北美肺癌发病人数22万人，死亡人数为15万人^[1, 2]^。在我国，每年因肺癌死亡的人数高达数十万人，2008年卫生部公布的居民死亡原因调查报告显示，肺癌已成为我国发病率和死亡率最高的恶性肿瘤之一。但肺癌的发病原因目前仍未完全明了，大多数研究认为与患者的遗传背景和后天环境因素有关^[3]^。遗传背景包括恶性肿瘤家族史、某些代谢酶基因多态性等^[4]^，后天因素主要与吸烟、环境污染等因素有关^[5-7]^。但肺癌发生的机制十分复杂，其确切发病分子机理仍不完全清楚^[8]^。因此，探寻肺癌的发生、发展、侵袭及转移的分子机制，为肺癌进一步治疗提供新的思路和新的靶点成为研究热点。近年来随着测序技术及分子生物学技术的不断发展，微小RNA成为研究的热点，其在表观遗传调控、肺癌细胞周期调控、基因的时空表达调控、细胞分化、细胞凋亡^[9]^等方面发挥重要作用^[10, 11]^。而且，微小RNA数量众多，其在肺癌中的作用机制多复杂^[12]^。本文对近年来关于微小RNA与肺癌关系的相关研究进行一简要综述。

## 1 微小RNA的发现

20世纪90年代，Lee及其同事首先发现秀丽杆线虫机体内存在有一种小分子RNA（lin-4），该小分子RNA并不编码任何蛋白质，但可以生成一对小的RNA转录本，每一个转录本能在翻译水平通过抑制一种核蛋白lin-14的表达而调节了线虫的幼虫发育进程。对上述现象的解释，大多数学者认为是因为lin-14编码的小分子RNA 3' 非编码区拥有特殊的重复序列和lin-4之间存在一定程度的互补结构所引起的。7年后研究者又发现了第二个microRNA-let-7，let-7相似于lin-4，也具有调节秀丽杆线虫生长发育的功能。此后，随着高通量测序、生物信息学等技术的发展，大量microRNA被陆续发现^[13]^。

## 2 microRNA产生机制

microRNAs的产生是由内源性基因编码并转录后产生的RNA转录本，此类RNA长度大多在21 nt-25 nt之间，其高级结构拥有短发卡特征。microRNA的产生开始于一个*microRNA*基因的pri-microRNA（primary microRNA）转录本（step 1）；这个70 nt-100 nt的发卡RNAs（pri-microRNA）在核内被核糖核酸酶Drosha加工处理而最终成为pre-microRNA（precursor microRNA，）（step 2）；pre-microRNA经由核转运蛋白送出细胞核（step 3），接着被第二个核糖核酸酶Dicer消化为21 nt-25 nt的microRNA（step 4）或在胞核被CAF（sDicer酶的同族物）加工成成熟的20 nt-24 nt microRNA；这个阶段的microRNA可以结合RISC（RNA-induced silencing complex，RISC可以将双链的microRNAs解离为单链）并与靶标mRNA互补并列（step5-6）；microRNA和靶序列的互补程度决定了靶基因mRNA是在在翻译水平被部分抑制，还是被完全断裂而降解（step 7）。植物体中，断裂似乎是主要的工作方式，而哺乳动物中则以翻译水平的抑制为主要抑制基因表达的机制。在人类，成熟的microRNA已被发现与Gemin3；Gemin4；EIF-C2等形成15S的核糖核蛋白体RNP复合物（也称RISC）。miRNP不仅能通过经典的microRNA途径作用于特异基因mRNA的3′非翻译区（3′-UTR），抑制mRNA的翻译而不影响mRNA的稳定性，也能RISC样降解与之完全互补的靶RNA^[14]^。近有报道^[15-17]^表明microRNA也作用于5′-UTR区，需要指出的是microRNA与3′-UTR和5′-UTR的结合是不完全互补的，但具体的机制仍不明确。

## 3 microRNA与肺癌的发生发展

研究^[18-20]^表明microRNAs与人肺癌的发生、发展存在着密切关系。众多动物实验和临床研究认为microRNAs可能是一种新的肿瘤发生发展调控因子。microRNAs既可发挥抑癌基因作用，下调原癌基因的表达；也可发挥癌基因的作用，下调抑癌基因的表达。如某些microRNAs在人非小细胞肺癌细胞内可发现其拷贝数显著增高，这类microRNA可能为癌基因所编码，它通过抑制细胞的分化和调亡，促进细胞增殖等方式来促进肺癌的发生与发展。如mir-17-92发挥了癌基因的作用，它是位于染色体的13q31的一个microRNA多顺反子，在肺癌患者的体内可以检测到其表达水平明显增高^[21]^。c-Myc是一个最具代表性的癌基因，它是一个螺旋—环—螺旋的亮氨酸拉链结构的转录因子，通过对10%-15%的基因进行调节从而影响细胞增殖、分化和调亡等。c-Myc的异常表达常常导致人类恶性肿瘤的发生。Donnell及其同事^[22]^指出c-Myc可同时激活E2F1和miR-17-92的转录，然而miR-17-92簇中的miR-17-5p和miR-20a抑制E2F1的翻译，故c-Myc调节miR-17-92和E2F1的表达，从而影响通过ARE-p53通路介导的细胞调亡，这种机制在肺癌的发生中也发挥重要作用。在人肺癌细胞中，很多microRNAs的拷贝数呈现明下降，编码此类microRNA的基因被称为抑癌基因。亦有研究发现，在肺癌中部分microRNA的直接靶基因为RAS，它通过3′UTR负性调节RAS的翻译表达^[23]^，从而影响肺癌细胞的分裂增殖。因此，相关研究认为microRNA let-7是通过调节RAS的机制在肺癌发生过程中起到抑癌基因的功能^[24]^。Takamizawa等^[25]^观察到microRNA let-7的表达水平在体内和体外的肺癌实验动物模型中都呈现低表达，microRNA let-7的低表达还与术后生存率的降低和肿瘤分期增高有着明显的相关性。他们发现microRNA let-7仅在肺癌中呈现低表达，而在乳腺癌和结肠癌中表达水平无明显降低。与microRNA let-7相反，miR-17-92的表达在肺癌中明显升高，其升高程度与肺癌的恶性程度呈现明显的相关性。Lewis等^[26]^通过相关实验证实了miR-17-92的靶基因包括*PTEN*和*RB2*两个抑癌基因。然而到目前为止，还未有实验证实这两个基因是真正属于miR-17-92簇。miR-17-92是否直接或通过靶向肺癌的抑癌基因参与肺癌的发生和发展仍然不是十分清楚。

## 4 microRNA与肺癌的侵袭和转移

转移通常指肿瘤细胞离开原发病灶，通过淋巴、血液、种植等途径，肿瘤细胞到达远处组织器官并形成新发病灶的过程。该过程发生发展是一个多基因参与的多步骤的复杂过程，且受到多重因素的影响和调节。新近的研究^[27, 28]^表明，microRNA在肺癌的远处转移中发挥关键性作用。这样的结论为研究肺癌发生、发展、侵袭、转移机制及其转移诊断和预后判断等方面提供了新的研究价值和思路。目前的研究认为，microRNA与肺癌侵袭转移相关性的机制主要表现为：①microRNA与上皮细胞间质转化（epithelial mesenchymal transitions, EMT）。EMT是指具有极性的上皮细胞转换成为具有活动能力的间质细胞并获得侵袭和迁移能力的过程，它通过改变细胞形态、减少细胞间粘附、重建细胞外基质、调节细胞内信号、抑制凋亡等多种方式，大大提高肺癌细胞的侵袭力和转移性。TGF-13途径是经典的EMT调节途径，研究发现miR-155、microRNA-200家族（miR-200a, miR-200b, miR-200c）均可能参与了EMT的调节^[29]^。②microRNA与肺癌细胞信号转导，在对microRNA与肺癌转移关系的研究中还发现它通过其他信号途径影响肿瘤转移。③miroRNA调节相关靶基因，microRNA-10b通过抑制HOXDl0，直接激活转移前基因RHOC，增加肿瘤转移可能^[30]^。

## 5 microRNA与肺癌诊断

随着microRNA在肺癌发生发展中的作用及其分子机制被逐渐发现，其在肺癌的诊断、治疗、分类、预后及风险评估方面逐步引起人们的关注。最近研究发现microRNA具有独特性和稳定性的特点，在实体瘤和血液肿瘤中均可检测到相关microRNA的异常表达；同时，通过对不同预后肺癌患者microRNA表达谱的分析，筛选出预后相关差异表达的microRNA作为预后相关标志物，评估其水平与患者预后的关系，建立肺癌预后microRNA模型^[31-33]^。相关研究通过检测肺癌患者和正常人的血清发现，miR-661、miR-411、miR-181b-5p和miR-486-5p在肺癌患者血清中的表达相对正常人群明显升高，并可作为肺癌诊断的血清学标志物，其联合影像学检查对肺癌的诊断效能明显提高^[34-36]^。

## 6 microRNA与肺癌治疗

microRNA的生物学功能决定其靶向治疗的可能性：①某些肺癌特异的microRNA低表达或者不表达；②某些肺癌特异的microRNA的高表达。对于前者可以采用外源基因导入的方法，提高其在肺癌中的表达水平，达到一致肺癌细胞的功能。但临床实践过程中，microRNA作为肺癌相关治疗方法并未得到应用。但其作为基因治疗的一种潜在手段已被相关动物实验证实。Du及其同事^[37]^通过向肺癌细胞中导入外源性小干扰RNA miR-93、miR-98及miR-197能够明显抑制肺癌细胞Fus1蛋白表达。因此，对于此类具有癌基因特点的microRNA，可通过外源性导入与其互补的其反义寡核苷酸来有效的降低其细胞内水平，从而达到抑制肺癌细胞的作用。

## 7 展望

尽管microRNA在肺癌发生机制、诊断及治疗中的重要性越来越受到广大科研工作者和临床医生的到关注，并且发现某些肺癌特异的microRNA能作为肺癌发生、发展的监测指标。但由于microRNA在肺癌治疗中存在的诸如脱靶效应，不能在体内持续稳定表达等问题，其在肺癌治疗方面的应用仍处在研究的初期阶段。到目前为止，microRNA的表达模式及生物学功能已部分得到阐明，但许多机制仍不完全清楚，相信随着研究的不断深入，microRNA在肺癌的诊断、监测、治疗与预防中的地位将日益显现^[38]^。
